# Bovine Piroplasma Populations in the Philippines Characterized Using Targeted Amplicon Deep Sequencing

**DOI:** 10.3390/microorganisms11102584

**Published:** 2023-10-18

**Authors:** Eloiza May Galon, Adrian Miki Macalanda, Tatsuki Sugi, Kyoko Hayashida, Naoko Kawai, Taishi Kidaka, Rochelle Haidee Ybañez, Paul Franck Adjou Moumouni, Aaron Edmond Ringo, Hang Li, Shengwei Ji, Junya Yamagishi, Adrian Ybañez, Xuenan Xuan

**Affiliations:** 1 National Research Center for Protozoan Diseases, Obihiro University of Agriculture and Veterinary Medicine, Obihiro 080-8555, Japan; eloiza.galon@cvsu.edu.ph (E.M.G.); rochelledybanez@gmail.com (R.H.Y.); chakirou82@yahoo.fr (P.F.A.M.); aringo2002@yahoo.com (A.E.R.); lihang-2020@hotmail.com (H.L.); jishengwei0903@hotmail.com (S.J.); 2 College of Veterinary Medicine and Biomedical Sciences, Cavite State University, Indang 4122, Philippines; amcmacalanda@cvsu.edu.ph; 3 International Institute for Zoonosis Control, Hokkaido University, Sapporo 001-0020, Japan; tatsuki.sugi@czc.hokudai.ac.jp (T.S.); kyouko-h@czc.hokudai.ac.jp (K.H.); kawai@czc.hokudai.ac.jp (N.K.); ktaishi@czc.hokudai.ac.jp (T.K.); junya@czc.hokudai.ac.jp (J.Y.); 4 Institute of Molecular Genetics, Parasitology, and Vector-Borne Diseases—Main Campus, Cebu Technological University, Cebu City 6000, Philippines; dr.adrianpybanez@gmail.com; 5 College of Veterinary Medicine—Barili Campus, Cebu Technological University, Barili 6036, Philippines

**Keywords:** piroplasma, amplicon sequencing, NGS, cattle, Philippines

## Abstract

Molecular assays and capillary electrophoresis sequencing have been used to identify parasites in livestock. The low sample capacity, which increases labor and processing time, is one drawback. Targeted amplicon sequencing (Ampliseq) uses the fast and large sample capacity platform to identify parasites in the target host, overcoming this limitation. DNA was extracted from 162 whole blood samples collected from cattle in three provinces in the Philippines. Using Illumina’s Miseq platform, the V4 hypervariable region of the piroplasma 18S rRNA gene was amplified and sequenced. The AMPtk pipeline was used to obtain distinct amplicon sequence variants (ASVs) and the NCBI BLAST non-redundant database was used to assign taxonomy. In total, 95 (58.64%) samples were positive for piroplasma. Using the AMPTk pipeline, 2179 ASVs were obtained. A total of 79 distinct ASVs were obtained after clustering and filtering, which belonged to genera *Babesia* (n = 58), *Theileria* (n = 17), *Hepatozoon* (n = 2), and *Sarcocystis* (n = 2). The ASV top hits were composed of 10 species: *Babesia bovis*, *B. bigemina*, *Theileria orientalis*, *Babesia* sp., *Hepatozoon canis*, *Sarcocystis cruzi*, *T. annulata*, *T. equi*, *T. mutans*, and *Theileria* sp. Thung Song. The results generated in this study demonstrated the applicability of Ampliseq in detecting piroplasmid parasites infecting cattle in the Philippines.

## 1. Introduction

Tick-borne diseases (TBDs) adversely impact animal health and cost farmers millions in income and opportunity losses worldwide. As such, prompt diagnosis is essential for the control and prevention of TBDs. The diagnosis of parasitic diseases, such as TBDs, has evolved as a result of molecular technology advances over the past half-century. Subsequently, molecular-based diagnosis has helped to elucidate the epidemiology of medically, veterinary, and economically important diseases [1]. Similarly, molecular techniques have been utilized to identify previously undiscovered pathogens [2,3,4]. 

Common traditional diagnostic tools for parasite detection may be performed through microscopy of blood smears or evaluation of the exposure of the animal to the parasite. However, microscopy is highly reliant on active infections and the skills of the microscopist, while serology depends on the presence of specific antibodies against the piroplasma parasites, which take time to develop in infected animals [5]. On the other hand, nucleic-acid-based diagnostic assays provide highly accurate detection of the agent in field samples, overcoming the various sensitivity and specificity issues of the former tools [5]. Therefore, it is anticipated that molecular tools will soon take the place of the more laborious and time-consuming conventional tools for parasite detection [3]. 

Molecular techniques, such as polymerase chain reaction (PCR) assays, have improved the surveillance and diagnosis of TBDs, particularly, that of babesiosis and theileriosis [3]. The widespread usage of molecular assays over traditional detection techniques is due to their superior performance in sensitivity and specificity, which attests to their outstanding field adaptability [4,5,6,7]. PCR assays with sequence-based characterization using classical sequencing platforms (e.g., capillary electrophoresis) have been the typical method for the identification of parasites via molecular detection [8]. Still, a significant bottleneck stems from the relatively laborious and expensive process that results from the limited sample throughput capacity [9]. The advent of the next-generation sequencing (NGS) era has made it convenient to fine-tune contemporary tools by streamlining the formerly challenging process. The recent drastic cost decrease of NGS has given rise to applications such as shotgun metagenomics, which analyzes the diversity of uncultured microorganisms in a sample using whole genomes [10], and marker gene sequencing, which uses a specific gene region to elucidate the particular microbial populations present in a given sample [11].

Targeted amplicon sequencing (Ampliseq), sometimes called marker gene sequencing, amplicon deep sequencing, or amplicon-based NGS, leverages the amplification of conserved genes with hypervariable regions (i.e., 16S rRNA, 18S rRNA, ITS) to determine the taxonomic classifications and identify phylogenetic relationships of microorganisms. It is a fast, high-throughput, and well-tested method for determining the phylogenies of microbes in a particular sample, including ones that are extensively contaminated with host DNA [12]. As a result, this technique has been used in several studies to examine the molecular epidemiology of economically important diseases such as TBDs. Notably, amplicon sequencing of piroplasma parasites has used the 18S rRNA gene as a marker gene in Cambodia [13], Pakistan [14,15], Thailand [16], and Zambia [17]. 

So far, piroplasmas that have been molecularly detected from cattle in the Philippines include *Babesia bovis*, *B. bigemina*, *B. naoakii*, *Theileria orientalis*, and *Theileria* sp. [18,19,20,21,22,23,24], most of which have been reported in the Luzon and Visayas regions. Of these, *B. bovis*, *B. bigemina*, and *T. orientalis* are among the most important piroplasma parasites in cattle in the Philippines, as they contribute to economic losses. PCR assays combined with Sanger sequencing analysis have hitherto been the only method used to identify piroplasma in livestock in the Philippines. Therefore, the purpose of this work was to evaluate the applicability of Ampliseq in the identification and characterization of bovine piroplasma populations in the Philippines.

## 2. Materials and Methods

### 2.1. Ethical Statements

Animal sampling procedures were performed following the *Philippine Animal Welfare Act* (Republic Act 10631) and the guidelines set by the Institutional Animal Care and Use Committee of the Cebu Technological University and Cavite State University. Experimental procedures and methodologies were approved by Obihiro University of Agriculture and Veterinary Medicine, Obihiro, Hokkaido, Japan (permits 20-128 and 1723-4). Before the start of sample collection, the animal owners were oriented and informed of the purpose of the survey and they provided consent to have their animals sampled.

### 2.2. Blood Sample Collection and DNA Extraction

**Figure 1:**
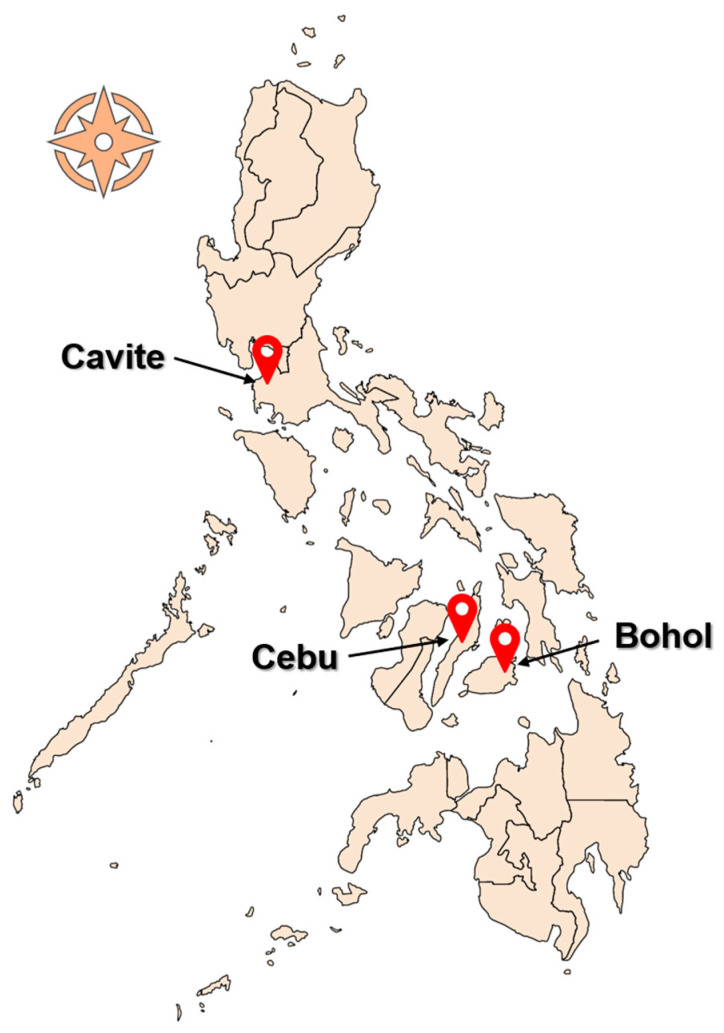
The Philippine map, showing the provinces where sampling was conducted.

In this study, whole blood (~2 mL) was collected from 162 cattle in three provinces in the Philippines. Sixty-two (n = 62), seventy-six (n = 76), and twenty-four (n = 24) samples were from Cavite, Cebu, and Bohol provinces, respectively (Figure 1). The sampling sites (backyard and stock farms) were chosen by convenience, while the sampled animals were selected randomly. Genomic DNA (gDNA) was isolated using the QIAmp DNA Blood Mini Kit (Qiagen, Hilden, Germany), following the protocols recommended by the manufacturer. Approximately 200 μL of whole blood was used and DNA was eluted in 100 μL elution buffer. To ensure the purity and quality, the DNA concentration of samples was assessed using a NanoDrop™ 2000 Spectrophotometer (Thermo Fisher Scientific, Waltham, MA, USA). Samples were stored at −20 °C until use.

### 2.3. PCR Assays for Screening and Amplicon Tagging and Multiplexing

The procedure and protocols of this study followed a previously published methodology [17]. The PCR designed for the reverse line blot (RLB) hybridization assay using primers RLB-F and RLB-R (Table 1) [25] was used to amplify the hypervariable V4 region of the 18S rRNA gene of piroplasma present in the bovine DNA samples. This test was selected because it met the optimum standard for tests to be employed in amplicon sequencing analysis: the binding of primers to highly conserved regions and amplification of a highly variable region [12]. The initial reaction was carried out in a final volume of 20 μL consisting of final volumes or concentrations of 12.9 μL UltraPure™ DNase/RNase-Free distilled water (Invitrogen, Waltham, MA, USA), 4 μM forward and reverse primers, 1× Ex Taq buffer (Takara Bio, Tokyo, Japan), 4 mM dNTP mixture, 0.5 U Ex Taq polymerase, and 3 μL gDNA. The PCR tests were run alongside appropriate positive (gDNA from *B. bovis* parasite culture) and negative controls (UltraPure™ DNase/RNase-Free distilled water). The thermocycling setup was performed at 94 °C for 5 min, followed by 35 cycles of 94 °C for 1 min, 50 °C for 1 min, and 72 °C for 1 min 30 s, and a final extension of 72 °C for 10 min. Amplification was verified by gel electrophoresis using 10 μL of the PCR product, ethidium bromide staining and viewing under UV light.

The remaining PCR products from positive samples were diluted 100 times with UltraPure™ water and underwent a 2-step PCR that attached Illumina tails and Illumina-index primers (metabarcodes) to the amplicons (Table 1). A reaction volume of 10 μL was used for the Illumina tail-tagging PCR, which included the following: 3.95 μL of UltraPure™ water, 2.5 μM of Illumina tailed-RLB primers, 5 μL of 2× Ampdirect^®^ Plus (Shimadzu, Kyoto, Japan), 0.25 U of BioTaq™ High Sensitivity DNA polymerase (Meridian Bioscience, Cincinnati, OH, USA), and 0.5 μL of 100-times-diluted PCR product. Except for the longer initial denaturation step (10 min) and the fewer amplification cycles (12 cycles), the cycling conditions followed a similar setup to the previous PCR settings. 

The high-fidelity KAPA Taq EXtra PCR kit (Kapa Biosystems, Wilmington, MA, USA) was used for the indexing PCR. The PCR components of the 20 μL reaction volume included 11.975 μL of UltraPure™ water, 0.25 μM each of Illumina-index primers, 4 μL of 5× buffer, 1.75 mM of MgCl_2_, 0.25 mM of dNTP mix, 0.675 U of KAPA Taq EXtra DNA polymerase, and 1 μL of 50-times-diluted PCR product from the tailing PCR. For the multiplexing of amplicons, eight forward (P5) and twelve reverse (P7) index primers were used for the 95 samples and 1 cultured *B. bovis* genomic DNA (positive control). The indexing PCR was run using the following thermocycling conditions: initial denaturation at 95 °C for 5 min, 15 cycles of denaturation at 92 °C for 30 s, annealing at 55 °C for 30 s, extension at 72 °C for 30 s, and a final extension step at 72 °C for 15 min. Then, 2 μL of indexing PCR product was electrophoresed on a 1% agarose gel stained with ethidium bromide, and viewed under UV light.

### 2.4. Library Preparation and Amplicon Sequencing

The amount of each sample used for sequencing was estimated based on the band intensity. Normalization of the indexing PCR products (2–10 μL per sample) was completed, and they were pooled into a library. After electrophoresis, the gel-suspended amplicon library was carefully excised and purified using Wizard^®^ SV Gel and PCR Clean-Up System (Promega, Madison, WI, USA). The amplicon library was sequenced in a 600-cycle format (2 × 300 bp) in an Illumina MiSeq platform (Illumina, San Diego, CA, USA) using a MiSeq Sequencing Reagent Kit v3 with a 25% PhiX DNA spike-in control, following the manufacturer’s instructions.

### 2.5. Bioinformatics and Phylogenetic Analysis

The acquired raw reads were demultiplexed and quality was checked with Trimmomatic [26] using the filtering criteria of TRAILING (20), SLIDINGWINDOW (4:15), and MINLEN (36). The AMPtk package [27] was used to generate amplicon sequence variants (ASVs) by concatenating both forward and reverse reads with a minimum merged length of 400 bp. These ASVs were denoised by DADA2 [28] and were filtered by LULU [29]. To assign taxonomy to the ASVs, the parameters used for the NCBI BLAST non-redundant (nr) database search were: -max_target_seqs 1, -perc_identity 70, -qcov_hsp_perc 70, and -evalue 1e-20 [30]. The obtained ASVs were clustered (criterion: 99% identity) using VSEARCH [31]. The workflow of the bioinformatics pipeline used in generating ASVs followed the study of [32], except for the searched parasite names (*Babesia*, *Theileria*, *Hepatozoon*, and *Sarcocytis* instead of *Trypanosoma*). ASVs with fewer than 10 reads per sample were judged invalid and were not included in further analysis [15].

Individual BLASTn searches were used to verify if the ASVs differed from those in the GenBank database. The ASVs of the same genus, except for *Babesia* for which a separate alignment was performed for each species, were aligned using Clustal W, and phylogeny was reconstructed using the maximum likelihood method and the best substitution model for phylogeny testing at 1000 bootstrap iterations in MEGA X [33]. The sequences obtained from this study were submitted to NCBI GenBank with accession numbers OR519725–OR519803 (284–520 bp).

## 3. Results

The RLB-PCR assay revealed that 95 of 162 (58.64%) gDNA cattle samples were positive for piroplasma. Cattle from Bohol had the highest detection rate (83.33%; 20/24), followed by those from Cavite (70.97%; 44/62), and Cebu (40.79%; 31/76). 

Following the use of DADA2, a total of 2179 ASVs were produced, 175 of which had their taxonomy determined using the BLASTnr database. Subsequently, 97 distinct ASVs were produced following dereplication, chimera removal, and clustering by VSEARCH. After discarding ASVs with fewer than 10 reads in each sample (n = 18), 79 ASVs were deemed valid. These ASVs corresponded to the genera *Babesia* (n = 58)*, Theileria* (n = 17)*, Hepatozoon* (n = 2)*,* and *Sarcocystis* (n = 2) (Table 2). The top taxonomy hits of the ASVs belonged to 10 species: *B. bovis* (n = 37), *B. bigemina* (n = 18), *T. orientalis* (n = 13), *Babesia* sp. (n = 3), *Hepatozoon canis* (n = 2), *Sarcocystis cruzi* (n = 2), *T. annulata* (n = 1), *T. equi* (n = 1), *T. mutans* (n = 1), and *Theileria* sp. Thung Song (n = 1). Table 2 depicts the most frequent piroplasmas detected in cattle by the number of sequence reads, namely *B. bovis* (259,704 reads), *B. bigemina* (207,232 reads)*,* and *T. orientalis* (347,535 reads).

**Figure 2:**
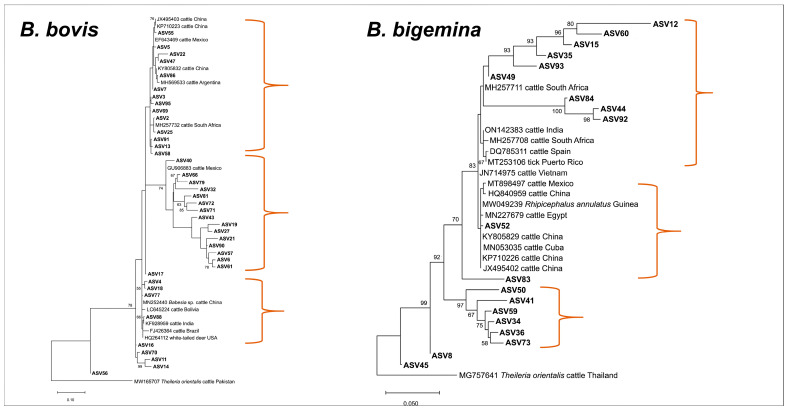
Phylogenetic trees of *Babesia* ASVs (413–434 bp). The tree was generated using the maximum likelihood method and Kimura-2 (*B. bovis*) and Tamura-3 (*B. bigemina*) parameter models, with a discrete Gamma distribution (4 categories (+G, parameter = 0.4708)) and a rate variation model for evolutionary invariability ((+I), 33.57% sites), and a discrete Gamma distribution (4 categories (+G, parameter = 0.4859)), respectively. The phylogeny inference was conducted using 1000 bootstraps. ASVs from the current study are shown in boldface. *Theileria orientalis* was designated the outgroup for both trees.

**Figure 3:**
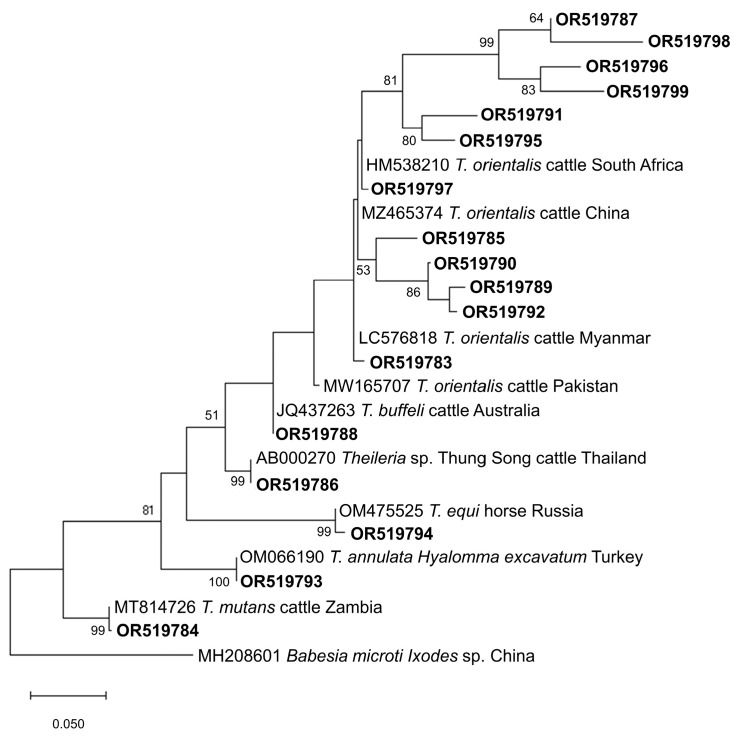
Phylogenetic tree of *Theileira* ASVs (284–462 bp). The tree was generated using the maximum likelihood method and Tamura-3 parameter model, with a discrete Gamma distribution (4 categories (+G, parameter = 0.3974)). The phylogeny inference was conducted using 1000 bootstraps. ASVs from the current study are shown in boldface. *Babesia microti* was designated the outgroup.

ASVs with top taxonomy hits of *B. bovis* (n = 37) and *Babesia* sp. (n = 2) were 90.76–100% identical with GenBank-deposited *B. bovis* isolates and showed 3 major subclades based on the generated phylogenetic tree (Figure 2). A total of 14 ASVs formed the first subclade, which clustered with *B. bovis* isolates from China (JX495403, KP710223, KY805832), South Africa (MH527732), Mexico (EF643469), and Argentina (MH569533). For the second distinct subclade, a total of 15 ASVs were found to be closely related to a bovine isolate from Mexico (GU906883), whereas the 4 ASVs in the third subclade grouped with isolates from China (MN252440), Bolivia (LC645224), India (KF928959), Brazil (FJ426364), and USA (HQ264112). Meanwhile, ASVs belonging to *B. bigemina* (n = 18) and *Babesia* sp. (n = 1) had 90.23–99.77% identity with isolates from the GenBank database and the phylogenetic tree indicated that the majority of the ASVs differed phylogenetically from previously reported *B. bigemina* isolates, with the exception of OR519775 (Figure 2). This is evidenced by the robust statistical values in the subclades. Phylogenetic analysis of *Theileria* ASVs revealed species-specific clustering (Figure 3). In total, 13 ASVs (91.72–100% identical to GenBank *T. orientalis* isolates) grouped with the *T. orientalis* complex subclade, and 4 of these ASVs assembled into a subgroup with strong nodal support. In addition, OR519786 and *Theileria* sp. Thung Song (AB000270) formed a sister clade to the *T. orientalis* complex subclade. As anticipated, ASVs taxonomically classified as *T. annulata*, *T. equi,* and *T. mutans* were grouped separately according to their respective species.

**Figure 4:**
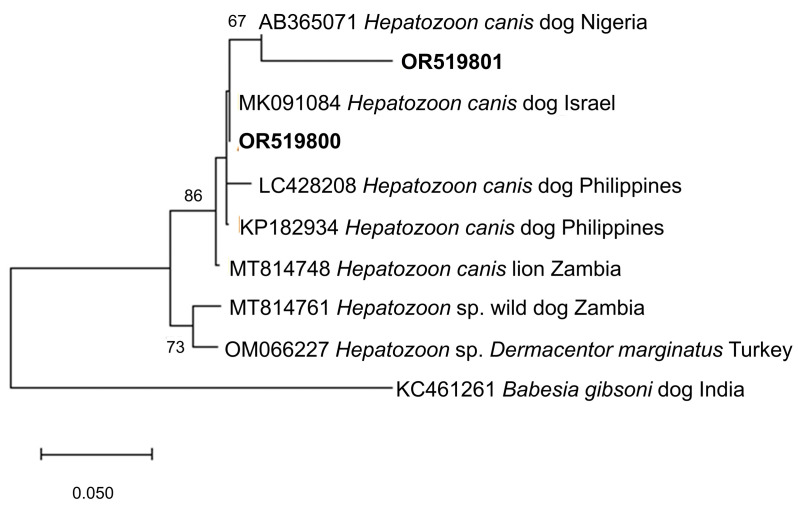
Phylogenetic tree of *Hepatozoon* ASVs (500–501 bp). The tree was generated using the maximum likelihood method and Tamura-3 parameter model, with a discrete Gamma distribution (4 categories (+G, parameter = 0.4047)). The phylogeny inference was conducted using 1000 bootstraps. ASVs from the current study are shown in boldface. *Babesia gibsoni* was designated the outgroup.

**Figure 5:**
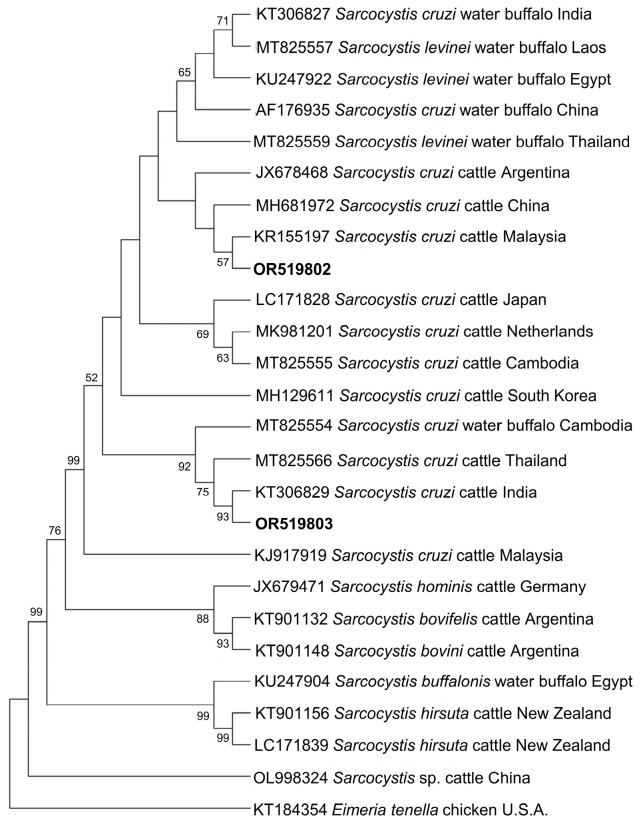
Phylogenetic tree of *Sarcocystis* ASVs (501–520 bp). The tree was generated using the maximum likelihood method and Tamura-3 parameter model, with a discrete Gamma distribution (4 categories (+G, parameter = 1.0235)). The phylogeny inference was conducted using 1000 bootstraps. ASVs from the current study are shown in boldface. *Eimeria tenella* was designated the outgroup.

Figure 4 and Figure 5 show the phylogenetic analyses of *Hepatozoon* and *Sarcocystis* ASVs, respectively. The two *Hepatozoon* ASVs were related to canine *H. canis* isolates from Nigeria (AB365071), Israel (MK091084), and previously detected Philippine canine *H. canis* isolates (LC428208 and KP182934) (Figure 4). On the other hand, the evolutionary inference of *Sarcocystis* ASVs revealed that OR519802 and OR519803 were most closely related to bovine *S. cruzi* isolates from Malaysia (KR155197) and India (KT306829), respectively (Figure 5). The grouping of OR519802 indicated a possible host-specific (cattle) subclade, while OR519803 suggested a geographical clustering (South and Southeast Asia) of isolates.

## 4. Discussion

Bovine herds face serious health risks from TBDs such as babesiosis and theileriosis. Millions of dollars are accounted as losses due to the impact that these diseases have on cattle farmers around the world. Bovine babesiosis and theileriosis are long believed to be endemic diseases in the Philippines due to the rarity of clinical cases despite the widespread presence of TBD agents. In the Philippines, previous molecular reports and species characterization have depended on the application of qualitative molecular techniques, i.e., the use of PCR assays and Sanger sequencing [18,19,20,21,24,34]. In the present study, we characterized the piroplasma populations circulating in field cattle raised in three provinces of the Philippines through the high-throughput NGS platform via the Ampliseq technique. 

More than half of the cattle samples examined (58.64%) were positive when tested using RLB primers, which is higher than a previous survey in four Philippine provinces that noted a 29.9% detection rate [24]. In contrast, using a similar assay, the rates reported in cattle from Zambia (63.4%) [17] and Pakistan (85.8%) [15] were higher than the rate of detection presented here. The identification of 79 ASVs belonging to four different parasite genera revealed the diverse parasite species in the infected cattle, providing new data on the tick-borne microbial communities in bovines in the Philippines.

The major causative agents of bovine babesiosis in the Philippines, *B. bovis* and *B. bigemina*, were the most commonly identified species in the cattle samples in the current survey. Using PCR tests, bovine *Babesia* species have been molecularly detected in at least 10 provinces nationwide [18,19,21,22,24,35]. *T. orientalis* was also present, which was similarly detected in previous surveys of bovines in the Philippines [20,21,24]. The species that causes oriental theileriosis, *T. orientalis*, which was previously believed to only produce mild infections, has been reported to be virulent and occasionally cause outbreaks due to the Chitose and Ikeda genotypes. [36]. Therefore, genotyping of the currently obtained *T. orientalis* isolates is recommended in future studies as it is expected to provide information into their potential to cause severe diseases, specifically in immunosuppressed and fatigued cattle [37]. 

Phylogenetic analyses identified several possible novel variants of *B. bovis*, *B. bigemina*, and *T. orientalis* based on the obtained sequences in the present study. This is consistent with findings in piroplasma populations in Pakistan, where new population variants or the emergence of cryptic species were suggested as possible explanations [15]. Given the relatively low percent identities (90–97%) of some ASVs with *Babesia* and *T. orientalis* sequences in the database, the latter hypothesis may be more plausible. However, due to the fact that only partial sequences were produced, caution should be exercised when identifying new population variants based on the 18S rRNA gene [14]. 

To the best of the authors’ knowledge, for the first time, the following species were molecularly identified in cattle in the Philippines: *T. annulata*, *Theileria* sp. Thung Song, *T. mutans, T. equi*, *H. canis*, and *S. cruzi.* The detection of *T. annulata* in the current study is a highly important finding because of its potential to cause severe disease and fatalities in cattle [38]. Additionally, this confirmation corroborates the detection of *T. annulata* in Philippine goats [39] and warrants more research as it can have catastrophic effects in the event of an outbreak. Meanwhile, *Theileria* sp. Thung Song and *T. mutans* cause mild infections in bovines [38,40]. *Theileria* sp. Thung Song was also detected in goats, as reported in a previous investigation in the Philippines [39]. The detection of *T. mutans* is unusual because it is known to be majorly present in Africa, but its detection here cannot be entirely dismissed as it was also found infecting swamp buffaloes in Malaysia [41]. This suggests that this species may be present in Southeast Asian countries.

Interestingly, *T. equi* and *H. canis* were identified and reported here, although both are non-conventional parasites of cattle. *T. equi*, one of the causative agents of the veterinary important disease equine piroplasmosis, is ubiquitous in horses in the Philippines [42]. Although equids are its natural and preferred hosts, *T. equi* has been found in other mammals such as dogs [43,44,45,46,47,48], camels [49], and tapirs [50]. The detection of *T. equi* in cattle (Algeria) has only been reported once [51]. This indicates that our knowledge of the host spectrum of *T. equi* is expanding and that cattle may play a role as possible reservoirs in the field. *T. equi* accidental transmission may also be a possibility for its detection. In the same manner, *H. canis*, a parasite transmitted by ingesting ticks, has been widely reported in dogs and cats in the Philippines [52,53,54,55,56], and *Rhipicephalus sanguineus* sensu lato is the confirmed tick vector [54,57]. One possible explanation of *H. canis* detection in cattle may be through accidental transmission via *Rh. microplus* ticks [58,59], the main tick ectoparasite of cattle in the Philippines. Technically not a piroplasma parasite, ASVs corresponding to *S. cruzi* were also obtained. The apicomplexan parasite *Sarcocystis* has a two-host life cycle [60]. Of the five *Sarcocystis* species that can infect cattle, *S. cruzi*, whose definitive hosts include dogs and wolves, is the most virulent and causes fever, anemia, hair loss, miscarriage, and stunting [61]. Previously, *S. cruzi* infection in cattle has been described in the Philippines [62,63], and its detection here emphasizes the importance of surveillance of other protozoan parasites in cattle.

## 5. Conclusions

The findings presented here demonstrated the applicability of the Ampliseq technique in characterizing the piroplasma populations of cattle in the Philippines. A relatively high diversity through several variants of *B. bovis, B. bigemina*, *Babesia* sp., and *T. orientalis* were recorded, and the possibly pathogenic *T. annulata* was molecularly detected in cattle for the first time in the country. Furthermore, *T. equi* and *H. canis,* both non-conventional parasites of cattle, were identified, and the first molecular identification of *S. cruzi* from blood samples in the Philippines was documented. Ampliseq may be adopted for the detection of other pathogens of economic importance in different hosts, leading to streamlined veterinary molecular diagnostics.

## Figures and Tables

**Table 1:** Sets of PCR primers used for amplicon sequencing in the current study.

Description	Primer Name	Primer Sequence (5′-3′)	Legend	References
V4 hypervariable region of the 18S rRNA gene of piroplasma	RLB-F	GAGGTAGTGACAAGAAATAACAATA	{RLB-F}	[25]
	RLB-R	TCTTCGATCCCCTAACTTTC	{RLB-R}	
RLB primers with Illumina tails	Illumina RLB-F	ACACTCTTTCCCTACACGACGCTCTTCCGATCT{RLB-F}	{IlluminaF}	[17]
	Illumina RLB-R	GTGACTGGAGTTCAGACGTGTGCTCTTCCGATCT{RLB-R}	{IlluminaR}	
Illumina-index primers	Illumina-i5	AATGATACGGCGACCACCGAGATCTACAC{index-i5 *}{IlluminaF}		
	Illumina-i7	CAAGCAGAAGACGGCATACGAGAT{index-i7 *}{IlluminaR}		

* represents index primers (D501-D508 and D701-D712).

**Table 2:** Top hits and composition of ASVs identified in this study.

Taxonomy Hit	No. of ASVs	Total Sequence Reads
*B. bovis*	37	259,704
*B. bigemina*	18	207,232
*Babesia* sp.	3	162
*T. orientalis*	13	347,535
*T. annulata*	1	459
*T. equi*	1	715
*T. mutans*	1	2188
*Theileria* sp. Thung Song	1	1100
*H. canis*	2	490
*S. cruzi*	2	818
Total	79	820,403

## Data Availability

The datasets generated during and/or analyzed during the current study are available from the corresponding author at a reasonable request.
